# Vulvovaginal Atrophy Following Treatment for Oncogynecologic Pathologies: Etiology, Epidemiology, Diagnosis, and Treatment Options

**DOI:** 10.3390/medicina60101584

**Published:** 2024-09-27

**Authors:** Ramunė Narutytė, Guoda Žukienė, Daiva Bartkevičienė

**Affiliations:** 1 Faculty of Medicine, Vilnius University, LT-03101 Vilnius, Lithuania; 2 Clinic of Obstetrics and Gynaecology, Faculty of Medicine, Institute of Clinical Medicine, Vilnius University, LT-03101 Vilnius, Lithuania; guoda.zukiene@mf.vu.lt (G.Ž.); daiva.bartkeviciene@mf.vu.lt (D.B.)

**Keywords:** vulvovaginal atrophy, cancer survivors, epidemiology, treatment

## Abstract

Vulvovaginal atrophy, characterized by the thinning of vaginal mucosa typically resulting from reduced estrogen levels, is frequently exacerbated by oncogynecologic treatments such as chemotherapy, hormonal therapy, radiotherapy, or surgery. This condition significantly impacts the quality of life for cancer survivors, leading to persistent discomfort, heightened infection risk, and negative effects on sexual function and self-esteem. Despite being a relatively common complication, vulvovaginal atrophy is not always discussed before the start of treatment. Treatments typically mirror those used for natural menopause; however, efficacy and safety data specific to this population are limited due to the exclusion of these patients from clinical trials. A major safety concern is the risk of hormone-sensitive cancer recurrence associated with estrogen therapy, which drives a preference for non-hormonal alternatives. Newer treatments, such as laser therapy, radiofrequency, and vaginal injections, show promise with minimal side effects and hormone-independent mechanisms, though efficacy data varies, highlighting the need for further research. This narrative review explores the epidemiology, risk factors, diagnosis, and management of vulvovaginal atrophy after the treatment for oncogynecologic disorders.

## 1. Introduction

Vulvovaginal atrophy (VVA) is the thinning of vaginal and vulvar tissues, commonly due to reduced estrogen levels, particularly in postmenopausal women when estrogen decreases by 95% [1]. Estrogen supports tissue health by promoting blood flow, thickness, elasticity, and secretions. Its reduction leads to tissue thinning, irritation, and potential bleeding during intercourse or gynecologic procedures [1,2]. Vaginal tissues normally contain glycogen, which lactobacilli convert into lactic acid, maintaining an acidic pH (3.5–5.0) that prevents pathogen colonization and infections [1]. Low estrogen reduces glycogen, decreases lactobacilli, disrupts vaginal acidity, and increases the risk of infection [1,2,3].

Symptoms include vaginal dryness, dyspareunia, irritation, itching, dysuria, urgent or frequent urination, recurrent urinary tract infections, and post-coital bleeding [4,5]. Untreated VVA can lead to vaginal or urinary tract infections and cause persistent abdominal pain or discomfort [5].

Recent literature suggests using the term genitourinary syndrome of menopause (GSM), which encompasses a broader spectrum of symptoms arising from lower urinary tract atrophy. GSM is described as a collection of symptoms caused by estrogen and other sex hormone deficiency in the major and minor labia, clitoris, vulva, vaginal vestibule, urethra, and bladder [4].

Patients with oncogynecologic diseases undergoing hormonal therapy, systemic chemotherapy, radiotherapy, or surgery experience ovarian damage or removal, leading to estrogen deficiency and the onset or worsening of VVA symptoms [2,3]. Hormonal therapy for hormone receptor-positive breast tumors includes aromatase inhibitors (AIs) for postmenopausal patients and selective estrogen receptor modulators, typically tamoxifen (TX), for premenopausal or low-risk postmenopausal patients [6]. AIs inhibit estrogen production, while TX’s metabolite, endoxifen, acts as an estrogen receptor antagonist but stimulates alpha estrogen receptors in the vagina, potentially increasing secretions and reducing VVA risk [3]. Chemotherapeutic drugs have gonadotoxic effects, causing follicular destruction and ovarian insufficiency [3,5]. Higher amenorrhea rates during treatment are linked to older age, positive hormone receptors, TX use, and anthracycline or taxane therapy [7]. Secondary menopause can also result from bilateral salpingo-oophorectomy, which is typically performed for endometrial or ovarian cancer or as a preventive measure for patients with BRCA 1 or 2 mutations [5]. Radiotherapy (RT) is employed in the treatment of cervical, endometrial, and vaginal cancer. RT not only has gonadotoxic effects but also disrupts vaginal tissue vascularization and innervation, leading to fibrosis [8,9]. This results in vaginal shortening and narrowing, with initial changes occurring within a few weeks and worsening over time [8].

Despite VVA being a common side effect, affecting 19–91% of patients treated for oncogynecological conditions [2,8,9,10,11,12,13], surveys indicate that only about half of oncologists discuss this complication before treatment, and 65–85% report lacking sufficient knowledge about VVA treatment options [2,14]. Discussing this complication in advance is beneficial not only for patient awareness but also for prevention purposes—local preparations applied before the onset of symptoms may reduce their intensity later on [15,16]. Furthermore, timely interventions not only relieve symptoms but also enhance the quality of life in cancer survivors [17,18].

Common treatments for VVA include vaginal lubricants and moisturizers, vaginal dilators, pelvic floor exercises, local estrogen and dehydroepiandrosterone, systemic hormone therapy, or ospemifene [5,19,20]. The efficacy of these methods is not always sufficient, necessitating the search for more effective treatments. New alternatives include treatments with hyaluronic acid or platelet-rich plasma (PRP) injections, laser, or radiofrequency treatment [1]. A clear understanding of treatment safety and efficacy in this patient group is vital for healthcare providers to choose the best approach, as some patients prefer other modalities over standard local therapies, improving adherence and outcomes [21]. VVA is a significant issue impacting women’s quality of life broadly. Ignoring symptoms can negatively affect not only patients’ well-being and health but also their sexual life, relationships, self-confidence, and self-esteem [1]. Breast cancer survivors experience earlier menopause and report more severe symptoms, such as vaginal dryness and dyspareunia, along with lifestyle limitations like avoiding physical activities. They also exhibit reduced emotional well-being, sexual function, and self-concept [22]. A study of patients treated with chemoradiotherapy found significant impairments in sexual activity and satisfaction. Most sexually active women reported limited enjoyment, 60% had major concerns about painful intercourse, and many experienced psychological effects, including reduced femininity and body image confidence [23]. Therefore, the aim of this review article is to examine the epidemiology and risk factors, diagnosis, and management of VVA following the treatment of gynecologic oncology disorders.

## 2. Epidemiology and Risk Factors

The prevalence of VVA among perimenopausal and postmenopausal women in the general population varies between 36.8% and 81.3% [24,25,26]. Key risk factors include age, menopause duration, vasomotor symptoms, and sedentary lifestyle [25,26]. Accurate prevalence comparisons between the general population and patients with oncogynecologic disorders are challenging due to factors like the inclusion of menopause clinic patients, who may be more likely to report VVA symptoms, potentially skewing results [24], and variability in diagnostic criteria, ranging from subjective symptoms to objective measures like pH or biopsy. Nonetheless, studies with control groups show that VVA is significantly more common in women with a history of oncogynecological conditions [8,10,24]. The prevalence and risk factors in different populations are shown in Table 1.

Frequency of VVA among patients treated with AI, TX, or chemotherapy ranges from 19% to 69.7% [10,11,28]. Patients receiving AI show higher rates of VVA compared to TX users (57.6% vs. 32.4%) and more frequently experience severe symptoms (33.3% vs. 5.95%) [10]. This tendency may be attributed to TX’s agonistic effects on vaginal estrogen receptors [10,28]. Contrary to the general population, younger AI-treated patients may be more likely to experience VVA, with one study reporting significantly higher prevalence in premenopausal compared to postmenopausal women receiving AI therapy (42% vs. 19%) [10].

The prevalence of vaginal toxicity following RT ranges from 30.7% to 91% [8,9,12,13]. Retrospective studies report varying frequencies of RT-induced vaginal toxicity, worsening over time. One study found 30.7% prevalence of mild stenosis 1.4 months post-treatment [12], while another reported 79% prevalence of moderate stenosis at 6 months [9]. Prospective studies show vaginal toxicity as early as 26 days post-RT, with an average onset at 9.6 months [13]. Cervical cancer patients show higher stenosis rates than those treated for endometrial cancer (79% vs. 67%), likely due to earlier diagnosis and less aggressive treatment of endometrial cancer [9]. The risk of vaginal stenosis in RT-treated patients is associated with age over 50 [13], combined chemoradiotherapy, higher radiation doses, external radiotherapy, and cervical adenocarcinoma, an aggressive histological subtype requiring intensified treatment [9].

## 3. Diagnosis

The diagnosis of VVA is typically based on patient complaints, medical history assessment, and examination of external genitalia and vaginal mucosa [2].

Most common complaints include vaginal dryness (64–100%) and dyspareunia (54.5–77.6%), followed by burning (38.3–61.7%), vulvar and vaginal itching (38.5–56.6%), and post-coital bleeding [24,25,26]. Urinary tract symptoms typically manifest later, including dysuria (12.6–36.1%), urgency or urinary tract infections [24,25,26,27]. However, some studies find that women diagnosed with VVA or those receiving hormonal cancer therapy experience urinary tract symptoms as frequently as women in control groups [10,11]. Symptom assessment is reliable for diagnosing VVA, with around 90% of symptomatic women confirmed objectively [27]. Conversely, not all patients with objective VVA signs report symptoms. One study found 53.1% of women had VVA signs on examination or *Papanicolaou* (PAP) smear, but only 34.9% reported symptoms [11]. Various questionnaires or scales are employed to assess symptom severity on a scale from 0 (no symptoms) to 3 (severe symptoms), often using a ten-point visual analog scale [29].

Sexual activity helps preserve vaginal epithelial integrity and flora; thus, avoiding it is discouraged [1]. Decreased sexual activity is also associated with a greater reduction in vaginal volume following RT [30]. The Female Sexual Function Index (FSFI) is frequently used in research to evaluate sexual function, measuring six domains: desire, arousal, lubrication, pain, orgasm, and satisfaction; a score above 26.55 is considered satisfactory [9,31].

Common vaginal examination signs of atrophy include mucosal dryness (60.3–99%), decreased rugosity (54.5–92.1%), pallor (47.4–90.7%), fragility (15.7–71.9%), and petechiae (15.1–46.7%) [25,26]. Reduced pubic hair, atrophy of the labia and clitoris, and hypopigmented spots on the external genitalia may also be evident [1,3,24]. Around 10% of patients with VVA might have symptoms without visible signs. Treatment is still advised in these cases, as VVA can progress and symptoms may appear before visible changes [24]. Vaginal pH measurement is an objective diagnostic tool, with VVA indicated by a pH > 5 [1,24].

The vaginal health index (VHI) can be used to objectively evaluate VVA by assessing elasticity, secretions, pH, petechiae, and moisture. Scores range from 5 to 25, with a score below 15 confirming VVA [29]. The VHI also correlates with the severity of vaginal dryness and FSFI scores [32].

The vaginal maturation index (VMI) evaluates estrogenization by analyzing epithelial cell types in a PAP smear. VVA is indicated by increased parabasal and intermediate cells and decreased superficial cells, with atrophy suggested if superficial cells make up less than 5% [24].

Although not standard in clinical practice, vaginal biopsies are performed in some studies to assess VVA. This approach allows for a detailed evaluation of epithelial thickness and dermal papillae, as well as an assessment of vascularity, innervation, and collagen expression through staining and morphometric analysis [8].

Despite variations in diagnostic methods and criteria across studies, researchers generally agree that VVA can be confirmed when the following three criteria are met: (1) vaginal pH > 5 and a decreased VMI, (2) subjective reports of vaginal dryness and/or other VVA symptoms, and (3) at least one objective sign of VVA (such as mucosal dryness, pallor, decreased rugosity, fragility, or petechiae) [25,26].

## 4. Treatment

Treatment options include non-hormonal and hormonal local therapies, pelvic floor muscle training, vaginal dilators, systemic hormonal treatments, ospemifene, and novel methods such as laser and radiofrequency therapy [28]. Safety and efficacy data for women with a history of gynecologic cancer are limited due to their exclusion from studies. Despite the high prevalence of VVA after cancer treatment, only 39.8% of breast cancer survivors with VVA receive treatment [12].

### 4.1. Local Non-Hormonal Therapies

First-line VVA treatment usually involves local preparations with high water retention capacity, often containing hyaluronic acid (HA). Low molecular weight HA penetrates well, while conjugated HA has prolonged efficacy due to slower degradation [16]. The results of studies investigating promising alternatives to HA, including topical treatments with polyacrylic acid [33], lactic acid [34], polycarbophil [35], aloe vera [36], chamomile [37], and vitamins D and E [38], are presented in Table 2.

Randomized controlled trials demonstrate that these local non-hormonal treatments positively affect both subjective (symptoms and FSFI) [15,33,34,35,36,37,38] and objective (VHI and VMI) [34,35,37,38] outcomes, with minimal side effects like local irritation [37] or vaginal discharge [36]. Some studies even suggest that non-estrogen treatments can achieve results similar to local estrogen. For example, six weeks of aloe vera or lactic acid gel use demonstrated comparable symptom relief [34,36], and a 12-week course of chamomile gel similarly improved FSFI scores [37]. Early initiation of local treatments can provide preventive benefits. One study found that suppositories containing HA, vitamins A and E started at the beginning of RT led to 90% of women reporting no or mild VVA symptoms, while 57.3% of the control group experienced moderate symptoms and 42.7% reported severe symptoms [15]. Oral vitamin D capsules have also been studied but showed no benefit over placebo for symptom relief or objective markers like VMI and pH [39].

Phytoestrogens, plant-derived compounds with weak estrogenic, anti-inflammatory, and antioxidant properties, are being explored as a treatment for VVA [5]. However, efficacy data remain insufficient. While some trials show benefits in VMI [40] and vaginal vascularization [40,41], most studies report placebo-like effects on symptoms, VHI, and pH [40,42,43].

Emerging local treatments like oxytocin and tamoxifen are promising. Studies report that daily use of oxytocin gel (300–400 IU) improves FSFI, VMI, pH, and epithelial thickness over 2–3 months without adverse effects [44,45]. One study found that oxytocin gel had a comparable effect on FSFI scores to promestriene cream, though promestriene was more effective at improving epithelial thickness [44]. Intravaginal tamoxifen (10–20 mg) shows minimal systemic absorption, reduces pH, relieves dryness, and lessens dyspareunia without significant short-term side effects [46]. However, further investigations are necessary to establish optimal dosing, safety, and efficacy of these treatments, especially in oncogynecologic patient populations not included in current studies [44,45,46].

### 4.2. Injections

HA can be used alone or with additives such as calcium hydroxyapatite, adipose tissue, PRP, and polynucleotides. Its effects last 3–6 months, with improvements noticeable within 1–2 months [47]. PRP injections alone also alleviate symptoms and can be combined with HA for enhanced results [48,49]. Single-arm clinical trials evaluating the efficacy and safety of injections are presented in Table 3.

HA and PRP injections improve VHI [48,49,50]. One study found this benefit, including symptom relief and improved FSFI, persisted for up to 6 months after a single injection [50]. Increased collagen gene expression was also observed, but there was no long-term effect on epithelial thickness [50].

HA gel injections may also protect the bladder and rectum during RT. One study reported that a 10 mL HA injection into the rectovaginal or vesicovaginal septum significantly reduced radiation exposure during brachytherapy [51]. HA injections have minimal side effects and require less frequent administration than other treatments [48,49]. Although a case of pulmonary artery thromboembolism has been reported, safety reviews have found no other serious adverse reactions [47].

### 4.3. Intravaginal Dehydroepiandrosterone

According to the North American Menopause Society, intravaginal dehydroepiandrosterone (DHEA) may be prescribed to women with hormone-dependent cancers when non-hormonal treatments are ineffective [19]. DHEA demonstrates positive effects on symptoms, pH, and VMI. It is converted to estrogens intracellularly, avoiding systemic estrogen elevation [19,29]. It has not been reported to significantly alter active sex hormone levels after 12 weeks of use [52] and does not affect endometrial cells [19,29]. However, since estrogen is a metabolite of DHEA, caution is advised when prescribing it to women with a history of hormone-sensitive cancer [19].

### 4.4. Ospemifene

The North American Menopause Society recommends ospemifene for moderate to severe VVA when local estrogen therapy is ineffective or unsuitable [19,20]. It promotes vaginal epithelial growth with minimal impact on other estrogen-sensitive tissues and is deemed safe after breast cancer treatment if a control mammogram is performed [29,53]. Contraindications include active hormone-dependent tumors and endometrial hyperplasia [19,53]. A meta-analysis found minor, clinically insignificant endometrial thickening (up to 1 mm) with no new cases of endometrial or breast cancer after 12–52 weeks of ospemifene treatment [54]. Additional research is required to confirm its safety in high-risk cancer populations; thus, its use should be determined by a multidisciplinary team in such cases [54].

### 4.5. Local Hormonal Therapy

According to the North American Menopause Society, local estrogen therapy may be an option following treatment for endometrial or breast cancer if non-hormonal approaches prove ineffective and oncologist approval is obtained [20]. A 2017 Italian survey found that only 15% of oncologists considered local estrogen therapy safe for breast cancer patients [2]. In contrast, a 2021 Australian survey showed much higher acceptance rates of 77% and 90%, depending on whether the cancer was hormone-dependent or not [14]. The primary safety concern for 71% of oncologists was the risk of cancer recurrence [2,14], highlighting the challenges in deciding on local estrogen therapy due to limited knowledge of its safety and current guidelines. Moreover, studies show no significant increase in hormone levels with 12 weeks of local estrogen use, even in women on hormonal breast cancer therapy [55,56]. Additionally, a 4.5-year cohort study involving 13,479 breast cancer survivors found that local estrogen therapy did not increase the risk of cancer recurrence [57]. Therefore, low-dose local estrogen therapy can be an option when other local treatments are ineffective [20,55,56].

### 4.6. Systemic Hormonal Therapy

Systemic estrogen therapy is generally avoided in women with a history of hormone-dependent cancer due to risks of breast cancer and endometrial hyperplasia, and is rarely prescribed to breast cancer survivors (about 3.5%) [2,20]. In contrast, approximately 79% of cervical cancer patients with vasomotor symptoms following chemoradiation receive systemic estrogen therapy [8]. Local estrogen therapy alone is less commonly prescribed after cervical cancer treatment (13–38%) due to its limited effectiveness [8,58]. However, it is theorized that vaginal fibrosis caused by RT restricts the delivery of systemic estrogen to epithelial receptors, highlighting potential advantages of local therapies [58].

### 4.7. Vaginal Dilators and Pelvic Floor Muscle Training

Vaginal dilators are especially important for patients with vaginal stenosis after RT, promoting tissue stretch and support, enhancing vaginal blood flow, and improving pelvic floor muscle control [1]. Usage guidelines vary: starting with 2–3 times per week or daily for 1–3 min, sessions lasting up to 10–30 min [30]. More frequent use shows greater benefits than longer single sessions. Initiation is recommended at least 4 weeks post-treatment, continuing 9–12 months; longer use may further reduce vaginal stenosis [59]. In a study comparing lubricants, local estrogens, testosterone, and dilators over 12 months, using dilators alone notably decreased symptom severity compared to other approaches [30].

Moreover, in a 12-week study, pelvic floor muscle exercises were found to improve VVA and urinary symptoms, as well as objective outcomes such as vaginal discharge, epithelial thickness, and mucosal color. These benefits were observed in women undergoing systemic hormone therapy as well [60].

### 4.8. Laser Therapy

Fractionated microablative carbon dioxide (CO_2_) and non-ablative Erbium YAG lasers are commonly used for VVA treatment. These lasers elevate the temperature of vaginal tissues to approximately 40 °C and cause microinjuries, promoting revascularization, collagen synthesis, and epithelial remodeling, thereby restoring tissue elasticity and moisture [1,29]. The standard treatment regimen typically involves three initial sessions spaced 30–40 days apart, followed by yearly maintenance therapy [29]. Table 4 shows the results of clinical trials assessing the efficacy and safety of laser therapy.

As shown in Table 4, studies indicate that laser treatment is more effective in improving subjective outcomes related to VVA, such as symptoms and FSFI, compared to objective outcomes like VHI, VMI, and epithelial thickness. However, data on the efficacy of laser therapy is ambiguous. Some clinical trials report no improvement in symptoms [64] or FSFI scores [66], or effects similar to placebo [61,67,70], while others show significant symptom reduction [68], comparable to hyaluronic acid [65] or estrogen therapies [63,71]. For objective markers, results also vary: Some studies show minimal impact on VHI [67,70], while others find it comparable to local hyaluronic acid [32] or estrogen therapies [66]. VMI findings are also inconsistent, with some studies showing no effect [71], while others indicate superiority over estrogen treatments [66]. Studies examining vaginal biopsies have not demonstrated favorable outcomes in terms of epithelial thickness, vaginal vascularization, and other histological parameters [63,69]. Nonetheless, most trials report at least one positive outcome [62,63,64,65,66,68,71], with no serious side effects. Minor reactions, such as discomfort, discharge [61,65,67,68,71], and one instance of urinary tract infection [71], have been noted. Studies involving post-gynecological oncology patients, mainly breast cancer survivors, often have small sample sizes and evaluate outcomes over 3–6 months [61,62,63,64,65]. Therefore, larger, longer-term studies with diverse populations are required to verify the effectiveness of laser treatment [61,62]. Moreover, studies that show no positive outcomes in postmenopausal women imply that laser treatment could potentially offer greater effectiveness for younger patients experiencing iatrogenic menopause [67].

### 4.9. Radiofrequency

Transcutaneous temperature-controlled radiofrequency and low-energy dynamic quadripolar radiofrequency are commonly used to treat VVA by promoting vaginal and vulvar tissue remodeling [29]. Table 5 shows the results of clinical trials assessing the efficacy and safety of radiofrequency.

Clinical trials have shown that radiofrequency improves VVA symptoms and FSFI scores within 3–4 months [63,72]. A 3-month study reported significant increases in VHI, comparable to estradiol cream, and positive effects on VMI [72]. Radiofrequency can be combined with other treatments for enhanced results; for example, combining four radiofrequency sessions with a single HA injection into the labia majora improved FSFI, symptoms, and VHI at 3 months [73]. Minor, short-term side effects include temporary burning, redness, pain, and irritation [63,72].

## 5. Limitations

This article provides insights into the epidemiology, risk factors, and potential treatments for vulvovaginal atrophy in women who have undergone treatment for oncogynecologic diseases; however, it has several limitations: Variability in diagnostic criteria for vulvovaginal atrophy leads to differing prevalence rates among cancer survivors across studies; Due to the lack of data on oncogynecologic cancer survivors, articles exploring treatment efficacy in healthy menopausal women were included; Most studies have a short follow-up period (up to 6 months); There is a notable absence of quality of life assessments; Most studies report clinical outcomes without incorporating histological or molecular analyses.

## 6. Conclusions

Oncogynecologic patients frequently experience vulvovaginal atrophy due to iatrogenic menopause and radiotherapy-induced vaginal toxicity. Risk factors include age, combined therapies, radiotherapy type, dosage, and aromatase inhibitor use. Diagnosis in clinical studies is typically based on symptoms, elevated vaginal pH, and a decreased Vaginal Maturation Index. While treatment approaches are similar to those used in natural menopause, the potential risk of hormone-sensitive cancer recurrence complicates the use of estrogen therapy. However, low-dose local estrogen preparations have proven to be safe. Emerging therapies, such as injections, laser and radiofrequency, show variable efficacy, with most studies reporting positive outcomes and indicating potential benefits from combined treatments. However, data on the efficacy and safety of standard treatment options for this specific patient population remain limited, emphasizing the need for further research to optimize and develop effective prevention and treatment strategies.

## Figures and Tables

**Table 1:** Prevalence and associated risk factors of VVA in different populations.

Authors, Year	Population	Prevalence of VVA and Vaginal Toxicity (Min–Max, %)	Risk Factors
Cagnacci et al., 2019 [25]; Palma et al., 2016 [26]; Palacios et al., 2018 [27];	Patients naturally experiencing menopause	36.8–81.3%	Older age [25] Menopause duration [25] Presence of vasomotor symptoms [25] Sedentary lifestyle [26] Malignant or benign breast pathology [27]
Cook et al., 2017 [11]; Baumgart et al., 2011 [10]; Biglia et al., 2003 [28];	Breast cancer patients treated with hormonal cancer therapy or chemotherapy	19–69.7%	Premenopausal status (compared to postmenopausal) [28] AI use (compared to TX) [10]
Hofsjo et al., 2018 [8]; Morais Siqueira et al., 2022 [9]; Dias et al., 2018 [12]; Brand et al., 2006 [13];	Cervical and endometrial cancer patients treated with radiotherapy	30.7–91%	Age > 50 y. [13] Cervical cancer (compared to endometrial cancer) [9] Cervical cancer adenocarcinoma type [9] Combined chemoradiotherapy [9] External RT [8] Higher RT doses [8]

VVA, vulvovaginal atrophy; RT, radiotherapy; AI, aromatase inhibitors; TX, tamoxifen.

**Table 2:** Results of studies evaluating local HA alternatives for VVA treatment.

Author, Year	Participants, Mean Age (Years)	Group (n), Intervention, Regimen, Study Duration	Outcome					Conclusions
			Symptoms	FSFI	VHI	VMI	Adverse Effects	
Juliato et al., 2016 [33]	BC patients with VVA symptoms, 50	n = 25, polyacrylic acid gel, 3 times/week, 1 month	+	++	-	-	-	Polyacrylic acid gel was superior to the lubricant in treating sexual dysfunction
		n = lubricant, before sexual intercourse, 1 month	+	+	-	-	-	
Garcia de Arriba et al., 2022 [34]	Patients with VVA, 61	n = 80, lactic acid cream, daily, 43 days	+	-	+	-	Mild-moderate 37.9%	The efficacy of hormone-free vaginal cream is non-inferior to that of estriol cream.
		n = 71, estriol 0.1% cream, 2 times per week, 43 days	+	-	++	-	Mild-moderate 54.1%	
Cagnacci et al., 2022 [35]	Patients with VVA, 50	n = 28, polycarbophil gel, 2 times per week, 30 days	+	+	+	++	None	Polycarbophil gel is non-inferior to HA gel for improving VVA outcomes
		n = 28, HA gel, 2 times per week, 30 days	+	+	+	x	None	
Poordast et al., 2021 [36]	Patients with VVA, 61	n = 30, aloe vera gel, daily for 2 weeks, then 3 times per week, 6 weeks	+	-	+	+	Discharge (n = 2)	Aloe vera can be safely used as a treatment option in VVA
		n = 30, estrogen cream, daily for 2 weeks, then 3 times per week, 6 weeks	+	-	+	+	Vaginal burning (n = 6)	
Bosak et al., 2022 [37]	Women with sexual dysfunction (FSFI ≤ 26.55), 54	n = 30, 5% chamomile gel, 2 weeks daily, then 2 times per week, 12 weeks	-	++	-	-	Vaginal burning (n = 2)	Chamomile gel improved sexual function similarly to estrogen cream
		n = 32, 5% estrogen cream, 2 weeks daily, then 2 times per week, 12 weeks	-	++	-	-	-	
		n = 27, placebo gel, 2 weeks daily, then 2 times per week, 12 weeks	-	+	-	-	Vaginal burning (n = 1)	
Keshavarzi et al., 2019 [38]	BC patients with VVA symptoms, 43	n = 32, vit. E suppository, daily, 8 weeks	++	-	-	++	-	Vitamin D and E vaginal suppositories improve VVA symptoms, VMI, and pH. Feasible non-hormonal alternative in BC patients
		n = 32, vit. D suppository, daily, 8 weeks	++	-	-	++	-	
		n = 32, placebo suppository, daily, 8 weeks	x	-	-	x	-	

+, a positive effect compared to baseline; ++, a positive effect compared to both baseline and the other group; -, not evaluated; x, no effect; BC, breast cancer; FSFI, female sexual function index; VMI, vaginal maturation index; VHI, vaginal health index; HA, hyaluronic acid.

**Table 3:** Results of single-arm clinical trials investigating injections for VVA treatment.

Author, Year	No of Patients	Participants, Mean Age (Years)	Intervention, Study Duration	Outcome	Adverse Effects	Conclusion
Hersant et al., 2018 [49]	20	BC patients, 61	One combined HA and PRP injection, 6 months	Improved VHI	None	Promising method for improving VVA in BC patients
Berreni et al., 2021 [50]	20	Patients with VVA, 58	One HA injection, 8 weeks	Improved symptom severity, FSFI, VHI, and collagen gene expression, no change in vaginal epithelial thickness	-	Vaginal injections improved VVA symptoms, sexual function, and were associated with collagen gene expression, suggesting stimulation of collagen formation
Saleh et al., 2022 [48]	47	Patients with VVA, 52	Two PRP injections a month apart, 2 months	Improved VHI and sexual function (VSQ)	None	PRP injections are as safe and effective as monotherapy for VVA

BC, breast cancer; VVA, vulvovaginal atrophy; FSFI, female sexual function index; VHI, vaginal health index; VSQ, vulvovaginal symptom questionnaire; HA, hyaluronic acid; PRP, platelet rich plasma.

**Table 4:** Results of studies assessing the efficacy and safety of laser therapy.

Authors, Year	Participants, Mean Age (Years)	Intervention Groups, Regimen, Study Duration	Outcome						
			Symptoms	FSFI	VHI	VMI	Epithelial Thickness	Adverse Effects	Conclusions
Mension et al., 2023 [61]	BC patients, 54	n = 35, CO_2_ laser, 5 procedures, monthly, 6 months	+	+	+	+	-	Mild: 45.7%, moderate: 11.4%	Vaginal laser therapy was safe but no more effective than sham laser in improving GSM outcomes, suggesting a lack of efficacy
		n = 37 sham laser, 5 procedures, monthly, 6 months	+	+	+	+	-	-	
Gold et al., 2022 [62]	BC patients, 54	n = 22, Erbium YAG laser, 2 procedures, monthly, 3 months	+	-	+	-	-	None	Both intravaginal laser and suppository treatment appear safe and efficient for treatment of urogenital atrophy in BC patients
		n = 21, HA suppository, daily for 10 days, then 2 times per week, 3 months	+	-	+	-	-	None	
Fernandes et al., 2022 [63]	BC patients, 52	n = 23, CO_2_ laser, 3 procedures, monthly, 4 months	+	-	-	-	X	No serious adverse effects	Laser, radiofrequency, and promestriene delivered comparable, significant symptom improvements among BC patients
		n = 21, radiofrequency, 3 procedures, monthly, 4 months	+	-	-	-	X		
		n = 26, promestriene cream, 3 times per week, 4 months	+	-	-	-	X		
Quick et al., 2020 [64]	Gynecologic cancer patients, 57	n = 10, CO_2_ laser, 3 procedures, monthly, 3 months	X	+	-	-	-	None	CO_2_ laser therapy is feasible in gynecologic cancer survivors, with preliminary evidence of safety and improvement in sexual function compared with sham treatment
		n = 8, sham laser, 3 procedures, monthly, 3 months	X	X	-	-	-	None	
Fidecicchi et al., 2020 [65]	BC patients, 60	n = 34, Erbium YAG laser, 3 procedures, monthly, 3 months	+	-	-	-	-	Warmth sensation during the procedure, vaginal discharge	Erbium YAG laser hyperstack treatment of the introitus and vestibulum offers greater improvement in superficial dyspareunia compared to standard laser protocols
		n = 34, Erbium YAG laser with a hyperstack protocol for vestibulum and introitus, 3 procedures, monthly, 3 months	++	-	-	-	-		
Politano et al., 2019 [66]	Patients with VVA, 57	n = 24, CO_2_ laser, 3 procedures, monthly, 14 weeks	-	X	++	++	-	None	CO_2_ laser therapy showed better short-term effects than those of promestriene or lubricant with respect to improving vaginal health
		n = 24, promestriene cream, 3 times per week, 14 weeks	-	X	+	+	-		
		n = 24, lubricant, during sexual intercourse	-	++	X	X	-		
Page et al., 2022 [67]	Patients with VVA, 57	n = 28, CO_2_ laser, 3 procedures, monthly, 18 months	+	+	X	-	-	No serious adverse effects, light spotting after the procedure	The CO_2_ laser treatment response was comparable to that of sham applications
		n = 29, sham laser, 3 procedures, monthly, 18 months	+	+	X	-	-		
Salvatore et al., 2021 [68]	Patients with VVA, 58	n = 28, CO_2_ laser, 3 procedures, monthly, 4 months	++	++	-	-	-	Irritation during the procedure	CO_2_ laser is superior to sham laser and could be proposed as an effective alternative treatment for GSM
		n = 30, sham laser, 3 procedures, monthly, 4 months	X	X	-	-	-	None	
Li et al., 2023 [69]	Postmenopausal women with VVA	n = 22, CO_2_ laser, 3 procedures, every 4–6 weeks, 6 months	-	-	-	-	X	-	Fractional CO_2_ laser is not significantly different from sham treatment
		n = 24, sham laser, 3 procedures, every 4–6 weeks, 6 months	-	-	-	-	X	-	
Cruff et al., 2021 [70]	Patients with VVA, 60	n = 11, CO_2_ laser, 3 procedures, every 6 weeks, 6 months	+	+	+	-	-	None	Improvements in both CO_2_ laser and sham-arms suggest a possible placebo contribution
		n = 12, sham laser, 3 procedures, every 6 weeks, 6 months	+	+	+	-	-	None	
Paraiso et al., 2020 [71]	Patients with VVA, 61	n = 30, CO_2_ laser, 3 procedures, every 6 weeks, 6 months	+	+	-	X	-	Pain (n = 1), spotting (n = 1), discharge (n = 1), UTI (n = 1)	CO_2_ vaginal laser and vaginal estrogen treatment resulted in similar improvement in genitourinary syndrome of menopause symptoms as well as sexual function
		n = 32, estrogen cream, daily for 2 weeks, then 3 times per week, 6 months	+	+	-	++	-	Spotting (n = 2), breast tenderness (n = 1), migraine (n = 1), pelvic pain (n = 1)	

+, a positive effect compared to baseline; ++, a positive effect compared to both baseline and the other group; -, not evaluated; X, no effect; BC, breast cancer; VVA, vulvovaginal atrophy; GSM, genitourinary syndrome of menopause; FSFI, female sexual function index; VMI, vaginal maturation index; VHI, vaginal health index; HA, hyaluronic acid.

**Table 5:** Results of clinical trials investigating radiofrequency for VVA treatment.

Author, Year	Participants, Mean Age (Years)	Group (n), Intervention, Regimen, Study Duration	Outcome					Conclusions
			Symptoms	FSFI	VHI	VMI	Adverse Effects	
Fernandes et al., 2023 [63]	BC patients, 52	n = 21, radiofrequency, 3 procedures, monthly, 4 months	+	-	-	-	No serious adverse events	Laser, radiofrequency, and promestriene delivered comparable, significant symptom improvements among BC patients
		n = 26, promestriene cream, 3 times per week, 4 months	+	-	-	-		
		n = 23, CO_2_ laser, 3 procedures, monthly, 4 months	+	-	-	-		
Sarmento et al., 2023 [72]	Patients with VVA, 57	n = 40, radiofrequency, 3 procedures, monthly, 3 months	-	++	+	+	Burning, redness, pain, and irritation lasting 3–5 days	Radiofrequency was comparable in efficacy to estrogen cream for treating VVA
		n = 40, estradiol cream, 3 times per week, 3 months	-	+	+	++	None	
		n = 40, control group	-	X	X	X	None	
Kolczewski et al., 2022 [73]	Patients with VVA, 53	n = 20, HA injection combined with 4 radiofrequency procedures, 3–4 days apart, 3 months	+	+	+	-	-	Radiofrequency can boost the efficacy of HA injections

+, a positive effect compared to baseline; ++, a positive effect compared to both baseline and the other group; -, not evaluated; X, no effect; BC, breast cancer; FSFI, female sexual function index; VMI, vaginal maturation index; VHI, vaginal health index; HA, hyaluronic acid; VVA, vulvovaginal atrophy.

## Data Availability

Data sharing is not applicable to this article.
